# Utilization of marketing automation tools for delivery of a faculty development curriculum

**DOI:** 10.12688/mep.20084.1

**Published:** 2024-03-27

**Authors:** Sarah H. Michael, Cody Brevik, Danielle T. Miller, Jessica Hitt-Laustsen, John L. Kendall

**Affiliations:** 1 Department of Emergency Medicine, University of Colorado School of Medicine, Aurora, CO, 80014, USA; 2 Distance Learning, Genesis Educational, Denver, CO, 80220, USA

**Keywords:** Faculty development, Email, Text message, Marketing automation platform, Digital marketing, Engagement

## Abstract

**Background:**

Physician clinical educators play important roles in teaching, providing feedback, and evaluating trainees, but they often have variable preparation and competing demands on their time that make universal participation in workshops, seminars, or short courses designed to foster these skillsets inefficient or impossible.

**Methods:**

We designed and implemented a 52-week synchronous curriculum designed to address faculty opportunities to improve teaching skills, feedback for residents and medical students, and evaluation skills, which were delivered using marketing automation tools, including text messaging and email. We evaluated the programmatic impact and feasibility of using the implementation science framework.

**Results:**

Over a 104-week evaluation period, there were at least 10,499 total content impressions and 4558 unique recipients, indicating the significant reach of this program to approximately 120 faculty members. Faculty engagement with continuing education materials remained stable or increased over the 2-year evaluation period, indicating that programs like ours can have sustainable impacts. Resident evaluations of faculty across the six key domains also improved after the implementation of the program.

**Conclusions:**

Our experience with digital marketing tools reflects that they can be used to deliver impactful curricular content to faculty for continuing educational purposes and that faculty can use these resources in a sustainable way. However, because of the incomplete reach with any single communication, this type of content delivery is not appropriate for isolation as a material of critical importance. More research is needed to identify the best practices and additional education-related uses of this technology.

## Introduction

Physician clinical faculty have important responsibilities for the provision of teaching, feedback, and evaluation to trainees, yet often have competing demands on their time and limited faculty development within these education domains. The Accreditation Council on Graduate Medical Education (ACGME), which oversees United States-based physician training, requires that residency programs offer faculty development but proffers little further guidance ^1^. In the absence of standardization, the preparedness of individual faculty members for their roles as clinical educators is multifactorial, but is often dependent on factors such as whether they had access to residents as teacher programming during training ^2^, the availability of opportunities at their own institution, their intrinsic motivation, schedule flexibility, and the impact of competing demands on their time ^3, 4^. Therefore, training programs are challenged to identify common opportunities for faculty improvement or to offer resources supporting faculty development ^4– 6^.

We performed a needs assessment for a curriculum to address variation in emergency medicine (EM) faculty preparation for clinical education among approximately 120 faculty members who work clinically at one of two emergency departments (EDs) affiliated with our residency program. A qualitative analysis of annual rotation evaluations completed by residents in two prior academic years identified three priorities for faculty development: clinical teaching, feedback, and evaluation. After these three faculty development content areas were identified, we performed a literature search for possible educational strategies to deploy to our faculty. Interventions designed to ensure that faculty groups like ours have access to clinical education resources frequently take the form of workshops, seminar series, and short courses but these formats have limitations that make their application to such large groups a challenge. First, the implementation of workshops and short courses requires significant expenditure of opportunity cost, especially on the part of the developers, whose time preparing for and delivering the content must be accounted for, as well as that of faculty participants who generally have significant competing demands on their time ^7^. Additionally, the implementation of workshops, short courses, and seminar series are often infrequent with variable faculty attendance due to competing faculty time demands, making it difficult to include all faculty members. This infrequency may also contribute to the accelerated decay of skill and knowledge ^8^ and risk creation of an institutional culture where emphasis on teaching feels cyclical rather than continuous and compliance-driven rather than part of the physician faculty professional identity. With an eye toward engagement, we sought content delivery modalities that were used regularly and were already accepted by the faculty.

Thus, we developed a robust faculty development curriculum targeting our needs assessment and coupled it with an email and text message content delivery strategy to provide high-quality, relevant, just-in-time resources and create a multi-institutional alignment around clinical teaching priorities and expectations. The resulting curriculum comprised just-in-time, synchronized content that allowed us to cover many relevant topics with relative depth while avoiding redundancy and creating a sense of perpetuity for the educational mission. A just-in-time strategy ^9^ focused on content delivery via email and text messages immediately prior to clinicians starting their clinical shifts, allowed cognitive priming, encouragement, and reinforcement immediately before applying teaching and assessment strategies to learners. Each faculty development installment included brief, evidence-based content relevant to clinical teaching, feedback and/or evaluation, links to additional resources, and a call to action. Emails were designed to be read in less than three minutes. Text messages were designed to reinforce the emailed content. 

## Methods

The implementation and evaluation of this curriculum were granted exempt status with a waiver of consent by the Colorado Multiple Institution Review Board, as is typical for educational program implementation and evaluation at our institution.

Emails were scheduled and sent using a marketing automation platform (MAP) ( Mailchimp; Atlanta, GA, USA; available as web-based software with a “freemium” subscription model) to the faculty every Sunday evening. MAPs allow content developers to easily create engaging materials that are viewable as a web page, actively manage audience contact, and estimate engagement among recipients. All faculty members were automatically enrolled to receive emails but were permitted to disenroll at any time without penalty. During the seven days following the content email, faculty members who were scheduled to work clinically with learners received a text message one hour before their shift start time. These messages included a brief reminder of the week’s content and a clickable link to the MAP-generated webpage for their just-in-time review to prime faculty members for teaching. Text messages were scheduled by a program administrator using third-party software ( TextMagic, San Francisco, CA, USA). All faculty members practicing clinically at the affiliated institutions were automatically enrolled in text messages but were notified in advance that they could opt out at any time without penalty. Email and text message content is archived and linked in the Data Availability section ^10^.

After 24 months, we downloaded a campaign report from the MAP, which includes the number of recipients, opening rate, click through rate, number of unique views, unsubscribe rate, and bounce rate for each email. The reports were downloaded to a spreadsheet platform (Excel for Mac v. 16.58; Microsoft Corporation; Redmond, WA, USA) and data intended for business purposes or deemed otherwise not useful, including revenue and number of orders, was excluded for ease of analysis. A similar report was also downloaded to determine the number of text messages sent. Engagement data is not available for text messages, except for opt-out rates. We then graphed the weekly opening rate and analyzed the data and program using an implementation science framework.

The RE-AIM implementation science framework was developed by Glasgow, *et al.* ^11^ for the National Institutes of Health, and is the most validated and used model for assessment of implementation. It is freely available for public use. The acronym stands for Reach, Effectiveness, Adoption, Implementation fidelity, and Maintenance. Although this framework was originally developed for use in public health interventions, it can also be an effective tool for examining the impact and sustainability of longitudinal educational programs given their ubiquity in public health initiatives.

Reach was defined as the number of individuals willing to participate in an intervention. To describe this impact, we report the number of individuals enrolled over time, opt-out rates, and number of unique content impressions generated by each dissemination modality.

Effectiveness is a measure of the effects of the intervention, which we assessed using faculty evaluation data generated by emergency medicine residents working with the participants.

Adoption reflects saturation of the intervention within the institution(s). We report the institutional support for continuing the program and the expansion of the program to include additional participants.

Implementation fidelity is a measure of how closely the implemented intervention resembles the planned intervention. We describe the tools and resources used to deliver the curriculum, barriers, and enablers. 

Maintenance refers to the sustainability of intervention. We describe ongoing programs and adaptations to improve sustainability.

## Results

### Reach

Over the first 24 months of the program (January 1, 2018, to December 31, 2019), a total of 120 academic physician faculty members at the primary sites received 11,560 weekly content emails and 9810 text messages ^10^. The primary sites were emergency departments in a county-supported urban hospital with an emergency medicine residency program and an emergency medicine residency-affiliated university hospital. The MAP reported that 4558 (39.4%) emails had been opened. The MAP considers emails “opened” when an invisible, single-pixel image loads, so users who do not fully load images when reading email, a common option for the participants’ institutionally supported email client, may inadvertently deflate the estimated rate. As such, it is difficult to ascertain a true impact in terms of content views, but the open rate determined by MAP is likely to be underestimated. Owing to technical limitations associated with text messaging software, it is not possible to know the text message read or click-through rates. The percentage of faculty members who access the content using this modality alone or in conjunction with email is therefore unknown.

**Figure 1.**
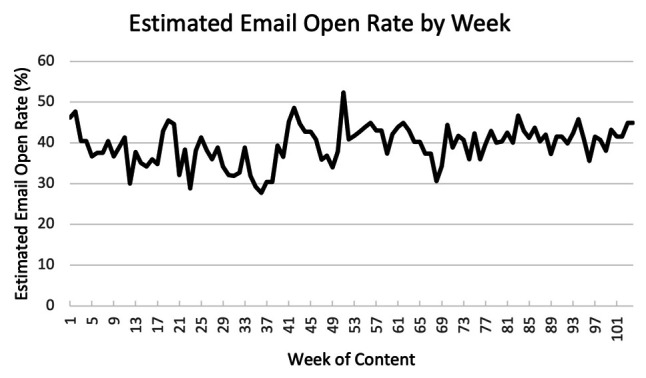
Estimated percentage of content emails viewed by faculty. The marketing automation platform-estimated email opening rate for each week of content demonstrated stable engagement (average 39.5%) during the 104-week evaluation period, ranging from 30.4% to 52.4%. It does not include emails that are open to text without images.

Despite the challenge of characterizing a true utilization rate, the technology and process utilized by the MAP to calculate it has not changed since the implementation of this program, and the rate itself has remained stable over time, averaging 38.5% (1059/2756) during months 1–6, 37.4% (1089/2932) during months 7–12, 40.5% (1135/2801) during months 13–18, and 41.6% (1275/3071) during months 19–24 ( Figure 1). One faculty member disenrolled from email and another 11 opted out of text messaging, but there was no overlap between the two groups, so all faculty members received the content through at least one modality during this time ^10^.

### Effectiveness

Quantitative metrics, including clinical teaching by faculty, faculty feedback, and faculty supervision, improved annually from to 2017–2019 on the ACGME resident survey after the curriculum implementation in 2017. Resident evaluations of faculty in several clinically relevant domains improved on annually required ACGME resident surveys after curriculum implementation ( Table 1). This data is provided by the ACGME and delivered aggregated for resident confidentiality, which limits further analysis. 

**Table 1.** Faculty teaching evaluation scores. Resident evaluations of the faculty for the year prior to implementation (2017) and the following two years showed improvement across six domains relevant to the curriculum delivered. All scores are on a 5-point scale.

		Assessment year			Percent change 2017–2019
		2017	2018	2019	
	Survey response rate	76% (51/67)	87% (58/67)	87% (58/67)	
**Domain**	Clinical teaching by faculty	3.38	3.28	3.52	4.1%
	Mentorship provided by faculty	3.17	3.19	3.34	5.4%
	Faculty supervision skills	3.48	3.43	3.64	4.6%
	Amount of supervision	3.56	3.48	3.81	7.0%
	Feedback provided by faculty	2.90	2.88	3.09	6.6%
	Resident-faculty interaction	3.33	3.40	3.62	8.7%

### Adoption

The program remains an important departmental faculty development initiative with administrative support and expansion of the program over time. In month 13, we adapted the curricular content for Advanced Practice Providers (APPs) (n=32), who worked in an affiliated ED and occasionally taught APP undergraduate and postgraduate trainees. In their first 12 months, the APP faculty had a MAP-calculated opening rate of 40.9% (569/1456) ^10^. We were subsequently approached by clinical faculty at an affiliated community hospital where EM residents rotate in the ED. Due to their less frequent shifts with residents, we used an opt-in procedure for this group, forwarding weekly emails for one month and then offering the opportunity to enroll. Of the 59 faculty members who could interact with residents at this institution, 33 (56%) opted for the program. In the first 6 months, the enrollees at the affiliate site had a MAP-calculated open rate of 58.2% (494/848) ^10^. Neither the community physicians nor APPs received text messages due to the administrative time that would be required to facilitate that process.

### Implementation fidelity

Using text messages and an MAP, we were able to implement a program with high fidelity to the curriculum we designed and sustained delivery of content using both modalities for 24 months. However, the costs of these tools vary. The use of a marketing automation platform ranges from free to more than USD 300/month depending on the size of the intended audience. Text messages can be scheduled and delivered at a small fee. Additional resources are needed to synchronize texts with a clinical or educational schedule, either via integration of another automated tool or through manual administrative time. The greatest enabler for a curriculum such as ours is the high-yield content that is relevant to the audience. Based on participant feedback, changes to the program after the initial 24-month implementation period included the development of an online content repository that allows faculty to quickly access relevant content and decreased text message frequency with an emphasis on the immediate relevancy of the just-in-time content. 

## Discussion

Our experience with digital marketing tools such as email and text messaging reflect that they can be used to deliver curricular content to faculty for continuing educational purposes and that faculty can use these resources in a sustainable way. At first glance, it is tempting to consider engagement with content as a relative failure because of open rates of 30.4%–52.4%. In their greater context, these rates reflect 10,499 total content impressions and 4558 unique recipient impressions, which, as noted above, likely represent an underestimation of the true impact. Organizing traditional faculty development programs or workshops to achieve content delivery of this magnitude, without marketing automation tools, would not have been feasible for our group.

While the cost of implementing such programs is not zero, curriculum developers may find that they offer outsized returns on investment when strategically employed. More recently, we found that the digital, synchronous content delivery we designed also offers important infection-prevention advantages in the context of the coronavirus pandemic. These strategies may supplement continuing education when opportunities to gather in groups are limited or undesirable. One limitation of using digital marketing tools to supplement other continuing education curricula is that the observed changes in the target audience’s performance are confounded by the presence of other curricula. Obviously, this could be scientifically mitigated with a randomized study design, which does not always align with the institutional goals related to content dissemination. While not addressed in this study, it may be feasible to employ an even broader application of these tools to the continuing education landscape. Theoretically, these tools can also be used to create institutional and organizational alignment around a variety of topics, including updated clinical recommendations and communication of upcoming or summarized educational programming. Additional studies exploring the potential roles of digital marketing tools in continuing education and faculty development would be welcome.

## Data Availability

Data underlying the results of resident evaluations of faculty teaching are available as part of the article, and no additional source data are required. They were presented as provided to the residency program by the ACGME. Open Science Foundation (OSF): Utilization of Marketing Automation Tools for Delivery of a Faculty Development Curriculum. https://doi.org/10.17605/OSF.IO/KJZ8U ^10^ This project contains the following underlying data: Primary Sites MAP Data.xlsx. (number of recipients, opening rates, and total content impressions among 120 emergency medicine residency physician faculty members over 104 weeks) APP MAP Data.xlsx. (number of recipients, opening rates, and total content impressions among 32 advanced practice provider faculties over 52 weeks) Community Site MAP Data.xlsx. (Number of recipients, opening rates, and total content impressions among 33 emergency medicine residency physician community faculty over 26 weeks) Open Science Foundation (OSF): Utilization of Marketing Automation Tools for Delivery of a Faculty Development Curriculum. https://doi.org/10.17605/OSF.IO/KJZ8U ^10^ This project contains the following extended data: Text Message Content.pdf (dates and content for text messages sent during 2018 calendar year) 2018 Email Content.pdf (email content with redactions of images and media available in the public domain or as web resources not owned by the authors and for participant privacy) Data are available under the terms of the Creative Commons Zero "No rights reserved" data waiver (CC0 1.0 Public domain dedication).
