# Supplementary material for: Utilization of marketing automation tools for delivery of a faculty development curriculum
Source: MedEdPublish (2016). 2024 Mar 27;14:19. [Version 1] doi: 10.12688/mep.20084.1 (PMC11153984; doi:10.12688/mep.20084.1)
Supplement: Peer review history

# Reviewer report: Reviewer response for version 1

**DOI:** 10.21956/mep.21507.r36838

**Published:** 2024-06-05

**Authors:** Michael Cassara

**Affiliations:** 1 Associate Professor of Emergency Medicine, Zucker School of Medicine, Northwell Health, New York, USA

Thank you for your submission to MedEdPublish. There are no actual or perceived conflicts on interest to report.

 Summary: The authors report on their development and implementation of an educational innovation (“synchronous” longitudinal “just-in-time” faculty development curriculum delivered by email and text message pushes using a marketing automated tool) to overcome barriers and challenges to content delivery using more traditional methods (CME courses, asynchronous on-demand learner-initiated content pulls, etc.). The intended audience for this faculty development curriculum were clinical faculty in emergency medicine located at two emergency departments in the United States. The authors’ aim of this faculty development curriculum was to enhance teaching, feedback, and learner assessment (presumably in the patient care environment). The authors gathered data largely centered on the metrics associated with the software (total number of email/texts sent, number/percent opened, disenrollments, etc.) and quantitative metrics aggregated from deidentified responses to items from the Accreditation Council for Graduate Medical Education (ACGME) annual resident survey concerned with the quantity and quality of faculty teaching.

 As I am interpreting this manuscript as an educational innovation, I am framing my review on the DoCTRINE guidelines as proposed by Blanco et al. (2022).

**Introduction**

 1. The authors provide an adequate description of the need and justification for the curriculum/innovation.The use of insights and data gained from a needs assessment/gap analysis is in keeping with curricular best practices (e.g., Thomas et al. 2022). This enhances attainment of the DoCTRINE criteria. Thank you. However, I find the author's’ use of the descriptors “synchronous” and “just-in-time” in the manuscript misleading. Synchronous, in my understanding of the term as applied to health professions education, commonly implies the implementation of an event “contemporaneous” with other learners (within a social learning context, whether the learning environment is a physical space or virtual milieu). A longitudinal series of posts to a discussion board, for example, is not usually referred to as “synchronous”. The authors appear to use this term to indicate that content pushes (by email and text) are occurring simultaneously to all recipients; “synchronized”, a term also used by the authors in the manuscript, seems more appropriate and provides the intended effect. I believe the distinction I am making is important: a simultaneously-received automated push does not guarantee or equate with the simultaneous opening of content nor does it represent a synchronous in-the-moment collaboration and social interaction with, by, and involving the content by multiple learners (whether co-locator or distributed). **Recommendation: I would not refer to this curriculum or its delivery method as “synchronous” but rather “synchronized”, “coordinated”, or “timed”.** Likewise, I find the phrase “just-in-time” has many meanings across education in general and specifically in health professions education. I had initially believed the authors’ use of the term more aligned with the thoughts discussed by Yilman et al (2021). After reading the Novak article and examining the authors’ content (especially the email content; see Reference 10, Michael et al. [2023] below), I better understood the alignment between this manuscript’s design and the concepts described by Novak (2011) (for readers of this review unfamiliar with Novak’s description, I would summarize by stating it embraces a “flipped classroom” approach to content dissemination that cognitively primes and prepares recipients in advance of clinical shifts to enhance student-student, student-teacher, and time on task when students and teachers are present together in the learning environment. **Recommendation: As the alignment of Novak’s “just-in-time” framework was not immediately understandable to me as a reader, I propose the authors consider reviewing the Novak framework in better detail and compare with other constructs of “just-in-time” (e.g., Yilman et al. [2021]); consider comparing and contrasting the two.**

 2. The authors provide an adequate review of the literature within the Introduction, including a recent Best Evidence in Medical Education report (systematic review on faculty development curricula by Steinert et al. [2016]). Later in the manuscript (Methods), the authors also incorporate a previously described and recognized framework to assess their technology-enhanced curricular implementation (RE-AIM). These examples support attainment of the DoCTRINE criterion. I acknowledge the limitations imposed by the brief report format. However, I believe there is room for the authors to provide 1-2 sentences of the theory/framework/model that informs on the Novak model of “just-in-time” and support the authors’ selection of pre-shift educator priming or knowledge dissemination via content pushes in favor of content pulls (see comment above and below this one). **Recommendation: To even further enhance fulfillment of the DoCTRINE criterion, the authors should better explain the theory that underlies the Novak framework/model to support why they chose it as part of their curricular design and implementation.**

 3. Unique contribution: The authors adequately describe how this this curricular innovation (utilization of the marketing automation tools to provides synchronized delivery of faculty development content) is unique from conventional methods of content dissemination for faculty development. They miss the opportunity to explain briefly how content pushes differ from content pulls. **Recommendation: To enhance fulfillment of the DoCTRINE criterion, the authors could compare and contrast their innovative method which involves scheduled pushes to its audience with others that leverage on-demand pulls from its audience (consider Orner et al. [2022]).**

**Curriculum development**

 4. The authors clearly state the purpose/goals of the educational innovation. Thank you.

 5. The authors imply that the objective of this curriculum is to enhance teaching, feedback, and learner assessment (presumably in the patient care environment). The authors do not provide specific detail for readers to understand exactly what learning outcomes (related to the faculty) are expected through engagement with this curriculum. Does the content build in a scaffolded way so that its intended recipients (the faculty as learners) grow developmentally as the longitudinal curriculum unfolds?  Readers would love to see the curricular map of how the content was scheduled to unfold (or was it random?). **Recommendation: While the authors provide supplemental digital access to their text messages/emails and the data, they do not provide a curriculum map that lists or describes the goals and objectives related to each faculty development content push by week. I believe this is feasible and would enhance attainment of this DoCTRINE criterion.**

 6. The authors clearly state the target audience of the educational innovation, supporting attainment of the DoCTRINE criterion. Thank you.

**Curriculum implementation and Results**

 7-8. The authors adequately describe the key features of the software and the resources needed to implement the curriculum, supporting attainment of the DoCTRINE criterion. Thank you.

 9. The authors adequately describe the instructional method. In the supplemental materials they provide through their registration with the Open Science Foundation (see Reference 10, Michael et al. [2023] below), the authors provide readers with access to the actual curricular tools, supporting attainment of the DoCTRINE criterion. Thank you.

 10. While the authors provide outcomes data on their implementation, most of their evaluation data center on program- and process-centered outcomes. The authors indeed provide adequate proof-of-concept to answer the question: does the marketing automated tool work to effectively disseminate information? Regarding the more important question of curricular efficacy, I do not feel the authors gathered sufficient evidence of curricular efficacy (as described in the RE-AIM) related to the learners. The responses to the ACGME resident survey are not specific enough. While the data is clearly depicted in the table and figure, the level of statistical analysis is minimal and descriptive. We are not truly able to infer correlation between this curriculum and any improvement of scores; clearly, causation is impossible. Even if the intent were to provide descriptive data, I believe the authors could have gathered more and provided a slightly more detailed level of analysis to support their story. They missed an opportunity to include mixed methods approaches to gathering richer data on curricular efficacy (e.g., self-reflection by faculty, free text comments by residents regarding change in the culture of clinical teaching/feedback, etc.). **Recommendation: Not sure if the authors are able to address this concern. If the authors have learner-specific outcomes (faculty outcomes, or indirectly, learner outcomes), they should include it to enhance attainment of this DoCTRINE criterion.**  

 11. The authors describe RE-AIM, one of their primary evaluation frameworks, supporting attainment of the DoCTRINE criterion. Thank you.

 12-14. The authors describe the number of participants. The authors provide raw data on the process- and program-centered outcomes(see Reference 10, Michael et al. [2023] below). See comment 10.

**DIscussion**

 15. The authors provide an adequate summary of findings, meeting the minimum expectations of the DoCTRINE criteria. However, I do believe they could have enhanced attainment of the DoCTRINE criteria for the Discussion section by expanding the discussion. I would have liked more reflective critique on the successes and opportunities they encountered over the period of implementation and observation. What happened during 2020-2022 with the experience of the pandemic? Finally, the authors could have provided more reflection and commentary, based on their experience, on the types of content (static v. dynamic, infographics v. video or audio media) that worked best using the Novak model and the marketing automated tool platform. They also could have provided more commentary comparing the push-pull aspects (as mentioned earlier).

Have any limitations of the research been acknowledged?

Partly

Is the study design appropriate and does the work have academic merit?

Partly

Is the work clearly and accurately presented and does it cite the current literature?

Partly

If applicable, is the statistical analysis and its interpretation appropriate?

No

Are all the source data underlying the results available to ensure full reproducibility?

Yes

Are the conclusions drawn adequately supported by the results?

Partly

Are sufficient details of methods and analysis provided to allow replication by others?

Yes

Reviewer Expertise:

health professions education, healthcare simulation

I confirm that I have read this submission and believe that I have an appropriate level of expertise to confirm that it is of an acceptable scientific standard, however I have significant reservations, as outlined above.

## References

[1] : The DoCTRINE Guidelines: Defined Criteria To Report INnovations in Education. *Acad Med* .2022;97(5) : 10.1097/ACM.0000000000004634 689-695 10.1097/ACM.0000000000004634 35171122

[2] : Just in Time Teaching (JiTT) Infographics App for Teacher Development. *J Grad Med Educ* .2022;14(3) : 10.4300/JGME-D-21-01011.1 344-345 10.4300/JGME-D-21-01011.1 35754631 PMC9200240

[3] : Utilization of marketing automation tools for delivery of a faculty development curriculum. *MedEdPublish* .2024;14: 10.12688/mep.20084.1 10.12688/mep.20084.1 PMC1115398438846581

[4] : Just-in-Time Teaching. *Routledge* .2009;

[5] : Curriculum development for medical education: A six-step approach. *Johns Hopkins University Press* .2015;

[6] : The Learning Loop: Conceptualizing Just-in-Time Faculty Development. *AEM Educ Train* .2022;6(1) : 10.1002/aet2.10722 e10722 10.1002/aet2.10722 35224408 PMC8848258

# Reviewer report: Reviewer response for version 1

**DOI:** 10.21956/mep.21507.r36837

**Published:** 2024-06-04

**Authors:** Rory Merritt

**Affiliations:** 1 Brown University, Providence, Rhode Island, USA

This article highlights challenges and potential solution to several common problem with faculty development (FD). Primary challenges in FD include lack of participant time, scheduling challenges, variability in content and unclear participation metrics. A potential solution proposed by the authors is to leverage marketing tools to develop bite-sized teaching information to EM faculty using weekly emails and pre-shift text messages. Results are somewhat limited but nonetheless important: number of total emails/texts sent and estimated email engagement. Results also include data related to teaching effectiveness. I am not a statistician but would like the know if the teaching effectiveness (resident ratings) are statistically significant. The use of an implementation science framework to support novel faculty development tools is important and this is a well-done paper.

Have any limitations of the research been acknowledged?

Yes

Is the study design appropriate and does the work have academic merit?

Yes

Is the work clearly and accurately presented and does it cite the current literature?

Yes

If applicable, is the statistical analysis and its interpretation appropriate?

Yes

Are all the source data underlying the results available to ensure full reproducibility?

Yes

Are the conclusions drawn adequately supported by the results?

Yes

Are sufficient details of methods and analysis provided to allow replication by others?

Yes

Reviewer Expertise:

Medical Education

I confirm that I have read this submission and believe that I have an appropriate level of expertise to confirm that it is of an acceptable scientific standard.

# Reviewer report: Reviewer response for version 1

**DOI:** 10.21956/mep.21507.r36841

**Published:** 2024-05-16

**Authors:** Giovanna Sirianni

**Affiliations:** 1 Clinical Public Health Division, University of Toronto, Toronto, Ontario, Canada; 2 Department of Family and Community Medicine, University of Toronto, Toronto, Ontario, Canada

Summary: This manuscript outlines a just-in-time faculty development intervention. This intervention included automated, email and text messages pushed to clinical supervisors weekly prior to working with learners. The messages included reminders of weekly content and links to faculty development materials. Outcome included number of emails opened. Engagement with text messages could not be determined. The authors suggest faculty teaching evaluation scores improved in the period of this intervention. Descriptive statistics provided for faculty teaching evaluations over a two-year period; statistical significance is not reported. The authors conclude that digital marketing tools (email/text) can be used to effectively deliver faculty development content. 

 Feedback:

 1) Faculty development is critical to support effective teaching, feedback and assessment in health professions education. The team should be commended on developing an intervention to support ongoing, just-in-time, faculty development to support clinical supervisors and to address commonly known barriers in this educational sphere. 

 2) Limitations of the study include:

 i) The introduction could be strengthened with more recent literature on the use of digital technology in the domain of faculty development. In addition, clarification of the 'needs assessment' would be helpful. It appears this was not a true needs assessment of faculty, but rather feedback on areas for improvement from the resident perspective. Changing the 'needs assessment' terminology would help clarify the data source that prompted the intervention. 

 i) The reach of the emails/texts reported does not equate with engagement/review/implementation of the content by the recipients. The study would be strengthened with quantitative and qualitative feedback from the clinical faculty on their engagement with the materials and how they applied them during supervision. This is an important piece of data that is missing. 

 2) The data presented on teaching evaluations includes descriptive statistics on percentage change over the study period. However, the team does not address if other faculty development interventions during the study period may have impacted these results. This section would be strengthened by addressing other factors that may have impacted scores and applying appropriate statistical tests to ensure the difference in scores is significant.

Have any limitations of the research been acknowledged?

Partly

Is the study design appropriate and does the work have academic merit?

Partly

Is the work clearly and accurately presented and does it cite the current literature?

Partly

If applicable, is the statistical analysis and its interpretation appropriate?

No

Are all the source data underlying the results available to ensure full reproducibility?

Yes

Are the conclusions drawn adequately supported by the results?

No

Are sufficient details of methods and analysis provided to allow replication by others?

Yes

Reviewer Expertise:

Faculty development, competency-based medical education

I confirm that I have read this submission and believe that I have an appropriate level of expertise to confirm that it is of an acceptable scientific standard, however I have significant reservations, as outlined above.
